# Keynote Address at the American Society of Breast Surgeons 18th Annual Meeting: Current and Future Application of Intraoperative Radiotherapy (IORT) in the Curative and Palliative Treatment of Breast Cancer

**DOI:** 10.1245/s10434-017-5942-z

**Published:** 2017-08-01

**Authors:** Frederik Wenz

**Affiliations:** Department of Radiation Oncology, University Medical Center Mannheim, Medical Faculty Mannheim, University of Heidelberg, Mannheim, Germany

## Abstract

Intraoperative radiotherapy (IORT) is increasingly used worldwide. Breast cancer is the most rapidly growing indication for IORT, approaching 70–80% of cases in most centers. This report reviews the theoretical background and clinical use of IORT for primary and metastasized breast cancer. There are established applications such as tumor bed boost during breast-conserving surgery followed by whole breast radiotherapy or IORT as a form of accelerated partial-breast irradiation (APBI) for selected patients. Novel applications such as IORT for vertebral or brain metastases are presented as well as technological developments, widening the spectrum of potential clinical applications for IORT.

The number of centers using intraoperative radiotherapy (IORT) worldwide has been steadily increasing during the last 15 years. During the 1990s, only about 30 centers treated mainly sarcomas, recurrent pelvic tumors, or locally advanced gastrointestinal cancer. The picture changed completely with the advent of IORT for breast cancer about the year 2000,^1^,^2^ novel mobile treatment units, and new knowledge as well as better understanding of the radiobiology of radiotherapeutic treatment with high single doses during the last 5 years.

## Radiobiologic Background^3^–^6^

Traditionally, radiation oncologists have always been reluctant to use single doses of more than 2–3 Gy. This reluctance is based on the fact that cells from different tissues in the petri dish have different shapes of the cell survival curves after radiation exposure. Normal tissue cells from late-responding organs (e.g., brain, breast, lung, liver) have low alpha/beta values and are assumed to be very sensitive to high single-dose treatment, yielding unacceptable chronic late reactions. It was common knowledge that tumor cells have high alpha/beta values (~10 Gy) and that fractionated treatment with doses of about 2 Gy per day during several weeks therefore result in a favorable therapeutic index with high tumour control probability (TCP) and low normal tissue complication probability (NTCP).

However, recent clinical data from brachytherapy and hypofractionation studies support the idea that tumors such as breast and prostate cancers have low alpha/beta ratios (~ 3 Gy) and would therefore not benefit from fractionated treatment. In addition, clinical experience using gamma knife radiosurgery and recently, stereotactic body radiotherapy (SBRT) or stereotactic ablative radiotherapy (SABR) demonstrates the safety and efficacy of high ablative radiotherapy doses when given to a small volume.

Knowledge about the molecular and cellular mechanisms of radiotherapy using high single doses has rapidly increased during the last few years. The concept of repair saturation, the influence on the cytokines in the microenvironment of the irradiated volume, and the interaction of cell death with the immune system have been understood only recently.

## IORT as a Boost for Breast Cancer

A local recurrence after breast-conserving therapy is a rare event, with far less than a 5% likelihood 5 years after high-quality treatment. However, for young patients with high-risk tumors, the risk for local recurrence can be considerably higher, defining the necessity to intensify local treatment by using escalation of the radiotherapeutic dose to the tumor bed (i.e., a boost for selected patients).

**Fig. 1:**
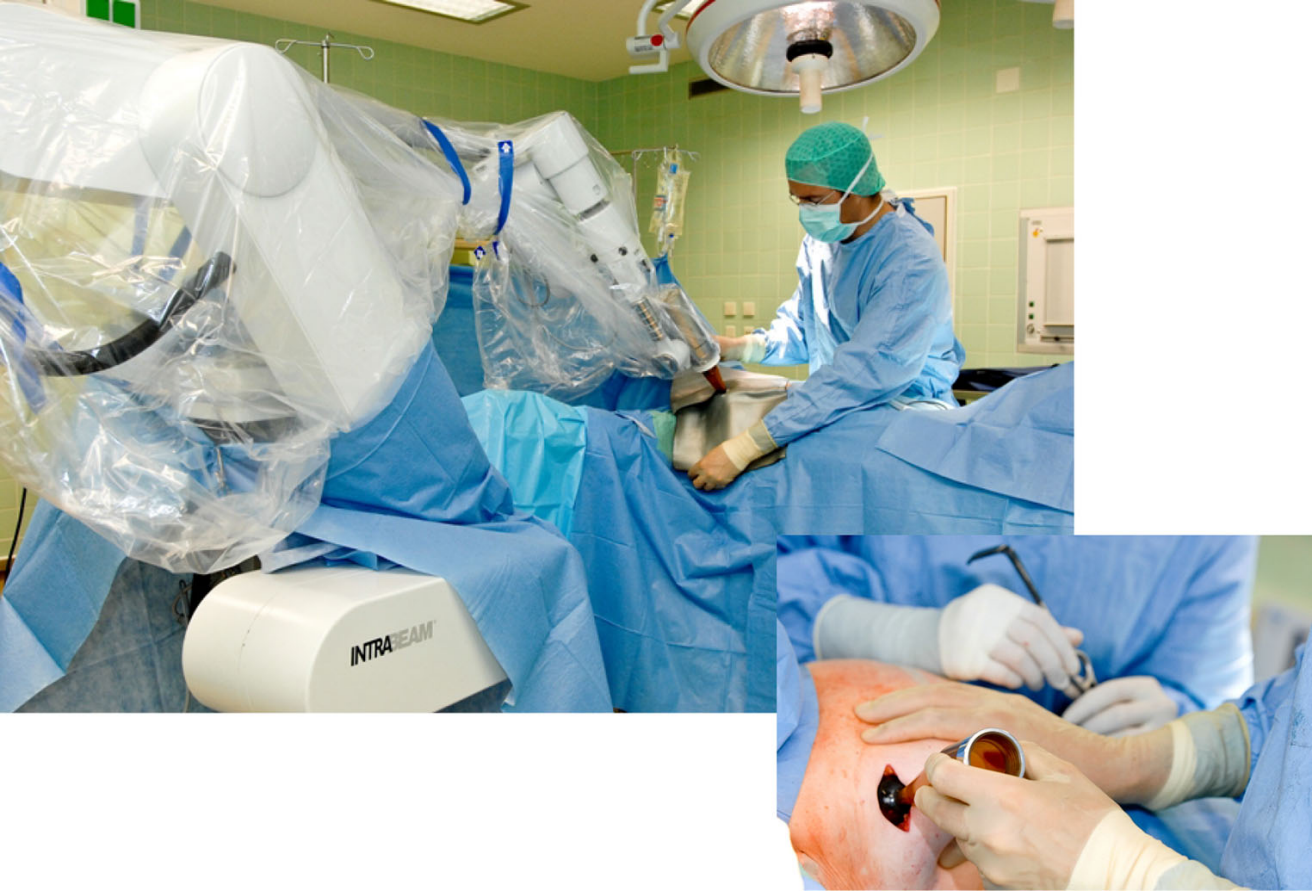
Intraoperative radiotherapy (IORT) during breast-conserving surgery

Proper definition of the target volume for boost treatment can be a challenge in clinical practice because most of the patients are seen by the radiation oncologist after completion of adjuvant chemotherapy and therefore months after surgery. The efficacy of the boost treatment can be limited for a considerable portion of patients by a geographic miss. Temporal miss is a novel concept based on the delay of radiotherapy and prolongation of the overall treatment time, which gives potentially remaining tumor cells time to proliferate, invade, and migrate in a stimulatory environment after surgical wounding.^7^ Geographic and temporal miss can be avoided by application of the tumor bed boost during breast-conserving surgery using IORT (i.e., applying the radiotherapeutic dose at the earliest possible time to the correct spatial point while also altering the cytokines in the microenvironment into a less stimulatory situation) (Fig. 1). Hence, most of the clinical series reporting outcome data after IORT boost treatment have cited extremely low local recurrence rates for cohorts of high-risk patients.^8^,^9^

## IORT as Accelerated Partial Breast Irradiation (APBI)

The concept of (accelerated) partial breast irradiation is based on the recurrence pattern of breast cancer after breast-conserving surgery, with most local recurrences found in or around the original tumor bed. All randomized clinical trials evaluating the complete omission of radiotherapy for selected low-risk patients have failed, yielding unacceptably high local recurrence rates of 4–5% or more after 5 years. Therefore, the concept of a shortened course of radiotherapy to a partial volume of the breast has been studied in several clinical studies.

To date, five prospective randomized trials have reported results, including two studies using single-dose IORT as the most accelerated form of APBI. All studies (Hungary, GEC ESTRO, TARGIT, ELIOT, Florence^10^–^15^) have reported non-inferior local recurrence rates for selected patients. A meta-analysis of the published survival data suggested even a potential benefit in the outcome in the partial breast irradiation (PBI) arms compared with the whole breast radiotherapy (WBRT) arms of the trials.^16^ All the trials consistently reported a reduction in radiation-induced side effects in the APBI arms.

Additional data are accumulating in further prospective studies or subgroup analyses (see Table 1 for selected examples: TARGIT A UMM,^17^ TARGIT E(lderly)^18^). Multiple national guidelines (e.g. ASTRO, ESTRO, German S3 guideline) recommend APBI as a treatment option for selected elderly low-risk patients. Depending on the selection criteria, the local demographics, the existence of a systematic screening programme, and other factors, about 15 to 30% of patients with a new diagnosis are eligible for APBI.^19^

**Table 1:** Additional current analyses of single-center data from the TARGIT A trial^17^ and from a prospective single-arm study (TARGIT E[lderly]^18^)

	PBI	WBRT
TARGIT A (UMM)	94.9% 5 year OS	92.7% 5 year OS
*n* = 185; med f/u 74 mo.	100% 5 year LRFS	98.8% 5 year LRFS
TARGIT E (> 70 yr)	98,6% 2.5 year OS	
n = 447; med f/u 14 mo.	99,4% 2.5 year LRFS	

*OS* overall survival, *LRFS* local relapse free survival, *PBI* partial breast irradiation, *WBRT* whole breast radiotherapy, *UMM* University Medical Center Mannheim, *f/u* follow-up, *yr* year

## IORT in the Palliative Treatment of Breast Cancer

**Fig. 2:**
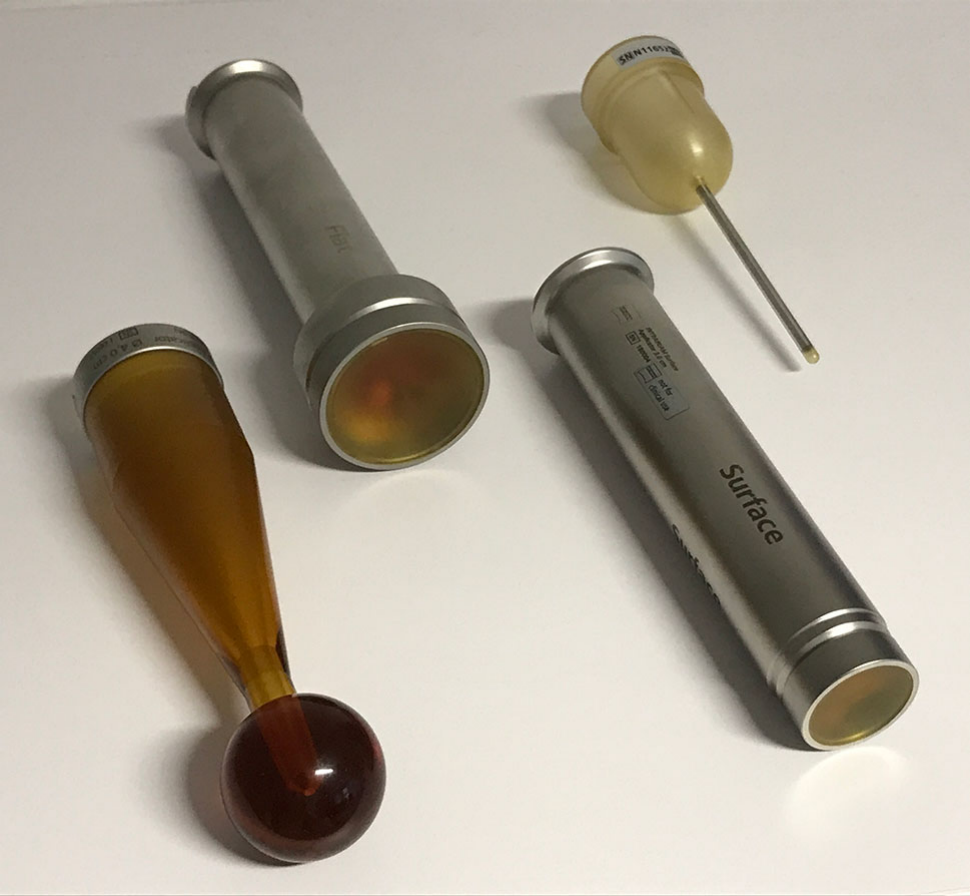
Technological developments including several new applicators. *Top left* flat applicator. *Top right* Kypho applicator. *Lower right* surface applicator. *Lower left* spherical applicator. These new applicators widen the treatment spectrum of intraoperative radiotherapy (IORT)

New mobile IORT devices, which can be moved from operating room to operating room, as well as the design and development of novel applicators allow a versatile application for IORT also in the palliative setting (Fig. 2). Besides individualized approaches, which are rather typical in the palliative, patient-centered treatment, systematic studies also are ongoing.

## Spinal Metastases (KYPHO–IORT)

Bone metastases are a frequent event in breast cancer, and most of the bone metastases are located in the spinal column. Treating physicians often are confronted in clinical practice with the therapeutic dilemma of breast cancer patients who experience simultaneous painful and potentially unstable spinal column metastases and progressive life-threatening visceral metastases.

**Fig. 3:**
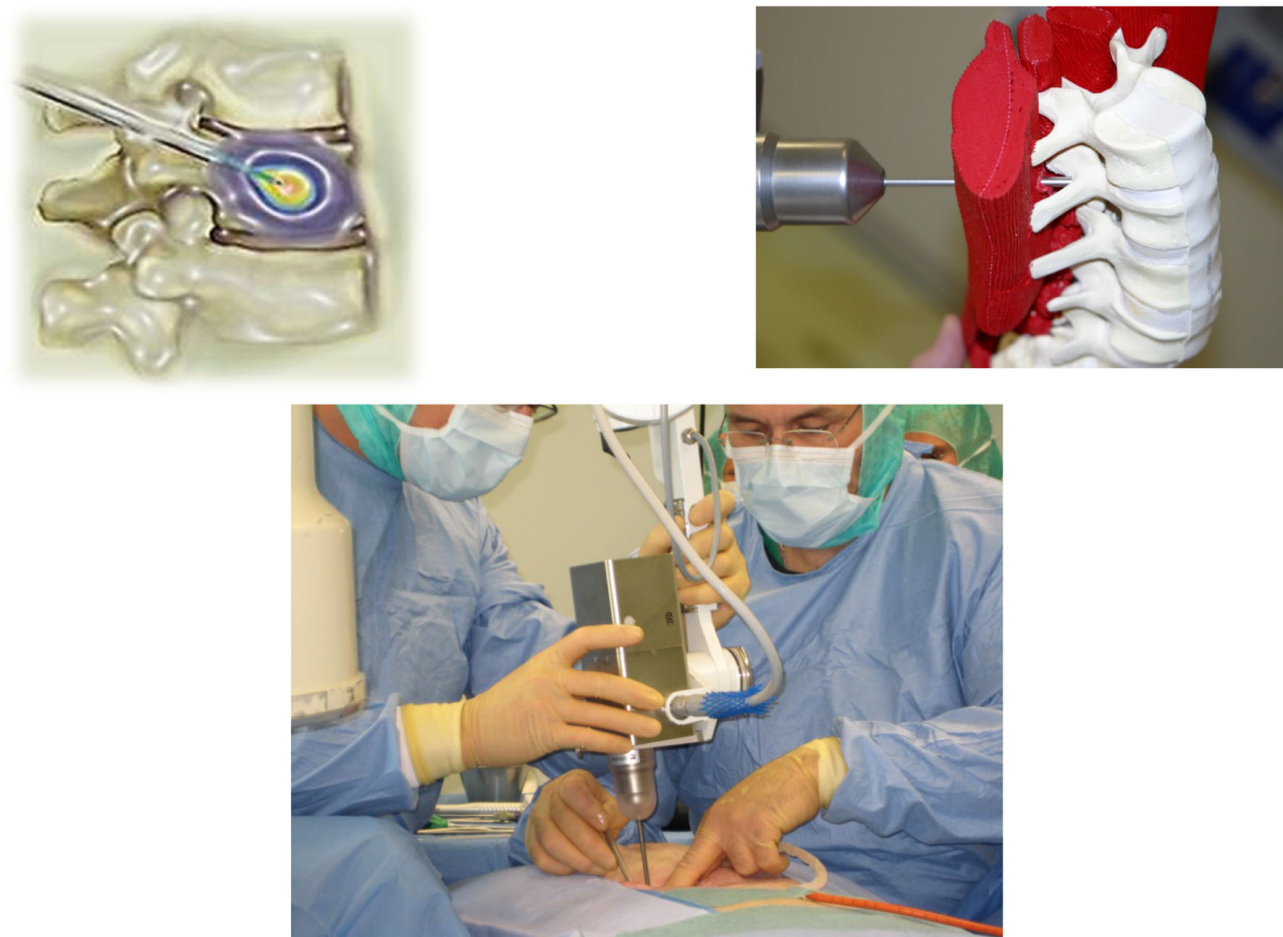
Kypho-IORT. A single dose of intraoperative radiotherapy (IORT) is given to the center of the vertebral metastasis during the kyphoplasty procedure

The challenge of optimal sequencing of therapeutic interventions (palliative pain reduction and preservation of mobility vs potential life prolongation by aggressive systemic therapy) frequently leads to controversial discussions on multidisciplinary tumor boards. The idea of a one-stop-shop minimally invasive intervention to achieve immediate stabilisation, fast pain reduction, and local tumor control led to the development of the KYPHO–IORT procedure (Fig. 3).^20^,^21^

Kyphoplasty per se is a patient-friendly intervention used increasingly, not only in the treatment of osteoporosis. Kyphoplasty stabilizes the vertebra immediately when the injected bone cement is hardened, in contrast to several weeks of recalcification after external beam radiotherapy. In addition, the microfractures, which cause a lot of movement-induced pain, are treated. However, kyphoplasty is not tumoricidal, so fast regrowth of the metastases can be expected. Adding a high single dose of IORT to the center of the metastases before injection of the bone cement sterilizes the tumor cells and prevents regrowth.

**Fig. 4:**
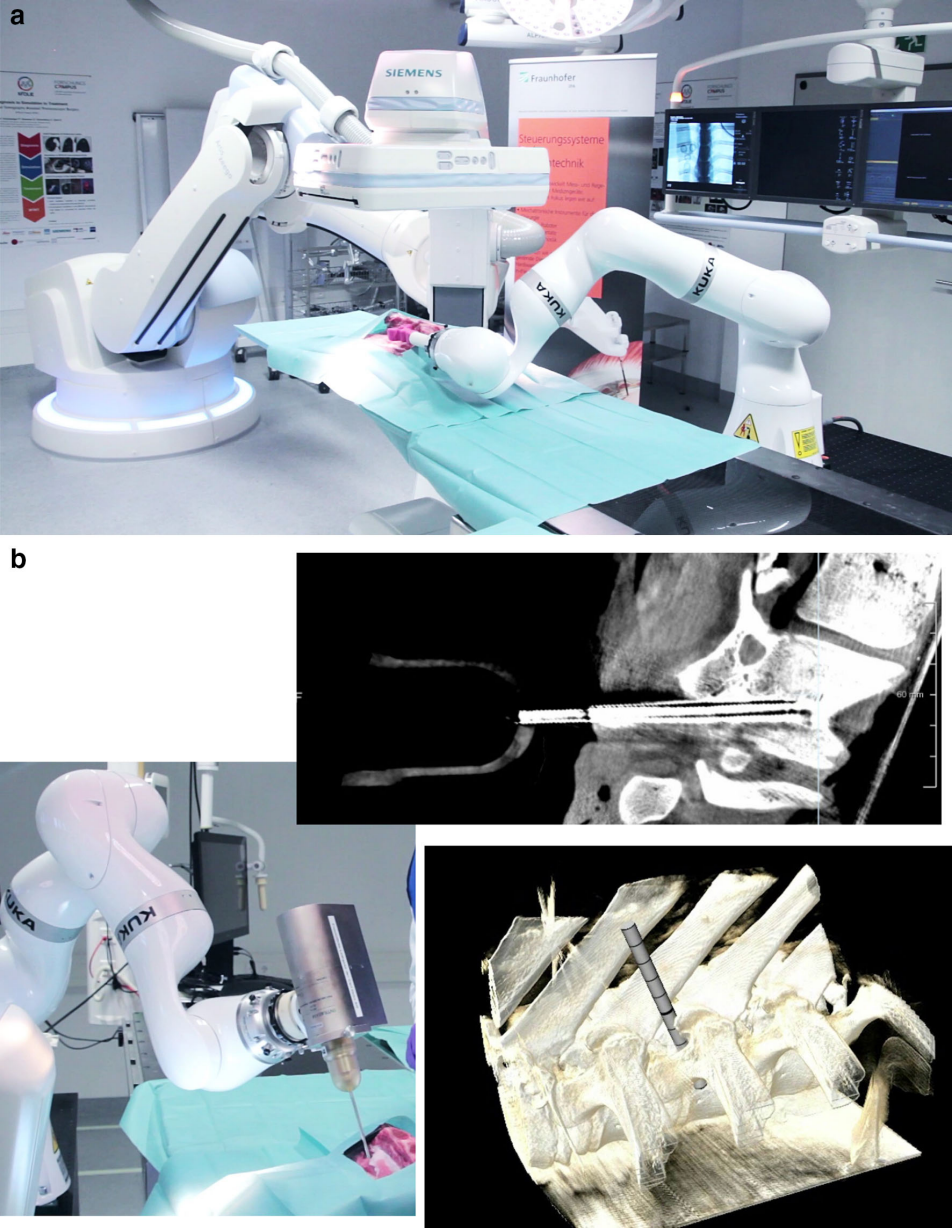
Robotic assistance and image guidance for intraoperative radiotherapy (IORT) is currently under development. **a** The robotic three-dimensional (3D) imaging system is registered with an assistance robot arm. **b** 3D imaging allows precise planning and guidance of the robotic-assisted procedure

About one-third of all patients with spinal metastases who present to the radiation oncologists are eligible for the KYPHO–IORT procedure.^21^ After completion of a phase 2 dose escalation trial (NCT01280032), about 100 patients have been treated, with a 1-year local control rate of 97%.^22^,^23^ A prospective randomized phase 3 trial (NCT02773966) is currently underway.^24^ In parallel, the technological development is ongoing within the framework of the research campus M^2^OLIE to implement robotic assistance and three-dimensional image guidance into the procedure (Fig. 4).^25^

## IORT for Brain Metastases

**Fig. 5:**
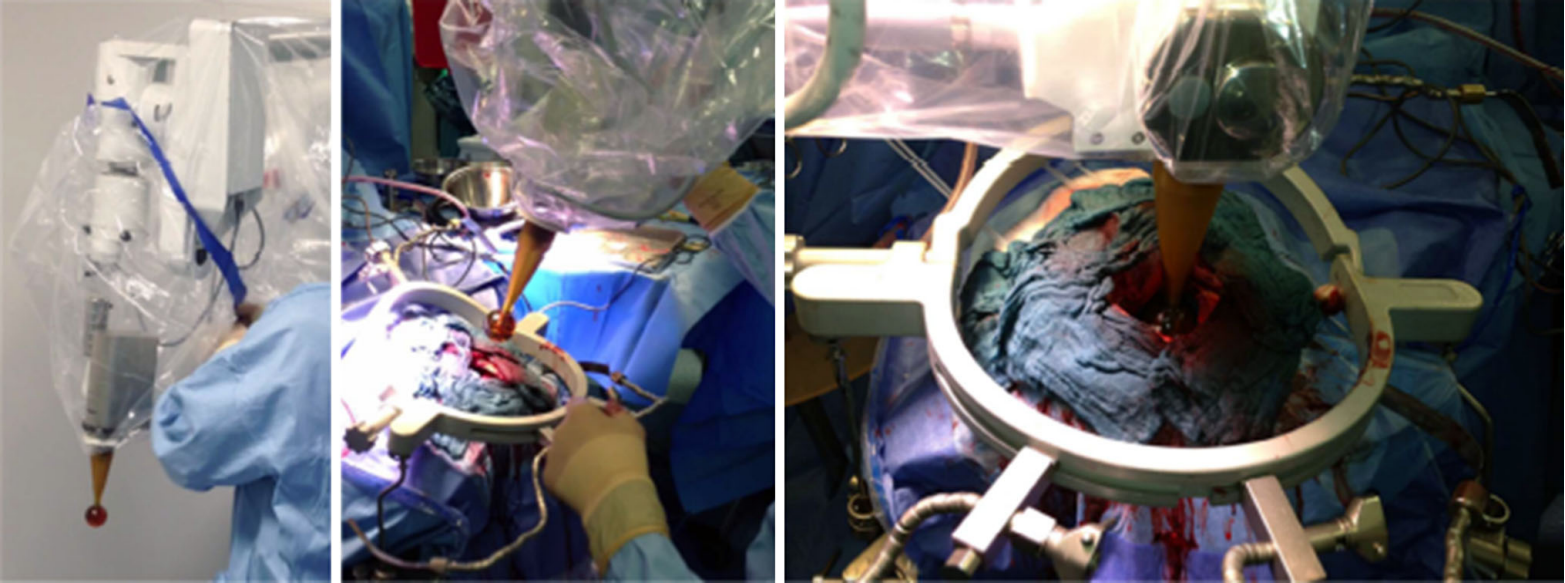
Intraoperative radiotherapy (IORT) for brain metastases during surgical resection

Postoperative whole-brain radiotherapy is the current standard of care after resection of brain metastases. Hair loss, fatigue, and other quality-of-life issues have led to the investigation of alternative concepts such as cavity-only radiosurgery for selected patients. Promising outcome data have already been reported with the use of gamma-knife or linac-based radiosurgery. Following the concept of temporal and geographic miss as well as the influence of radiotherapy on the microenvironment after surgical wounding (see earlier discussion^7^), a protocol for intraoperative cavity boost during surgical resection of brain metastases has been established (Fig. 5). A prospective clinical trial is under development.

## IORT for Recurrent Breast Cancer

Fortunately, local recurrence rates after breast-conserving surgery are extremely low. However, due to the high number of primary patients, the absolute number is still considerable. In addition, many patients have received radiotherapy to the breast previously (e.g., long-term survivors after Hodgkin’s disease). Mastectomy has been the standard of care in these cases of recurrent or secondary breast cancer due to the expectation of unacceptable side effects after a second course of WBRT.

Several small patient series have been published demonstrating the value of a second breast-conserving approach with tumorectomy and intra- or postoperative partial-breast radiotherapy.^26^–^30^ Local control rates are promising, and the rate of long-term side effects has been acceptable, which has led to the inclusion of this treatment option into national guidelines.

## Summary

The use of IORT has steadily increased during the last 15–20 years and currently is clinically established in more than 300 centers. The curative and palliative treatment of breast cancer is one of the most frequent indications for IORT, accounting for up to 75% of the cases worldwide. Accumulating clinical data emphasize the clinical value of IORT, and the current technological development suggests even more clinical use in the future.
